# Hospitalisation rates differed by city district and ethnicity during the first wave of COVID-19 in Amsterdam, The Netherlands

**DOI:** 10.1186/s12889-021-11782-w

**Published:** 2021-09-22

**Authors:** Liza Coyer, Elke Wynberg, Marcel Buster, Camiel Wijffels, Maria Prins, Anja Schreijer, Yvonne T. H. P. van Duijnhoven, Alje P. van Dam, Mariken van der Lubben, Tjalling Leenstra

**Affiliations:** 1 Department of Infectious Diseases, Public Health Service of Amsterdam, Nieuwe Achtergracht 100, 1018 WT Amsterdam, The Netherlands; 2 Department of Infectious Diseases, Amsterdam institute for Infection and Immunity (AII), Amsterdam UMC, University of Amsterdam, Amsterdam, The Netherlands; 3 Department of Epidemiology, Health Promotion and Care Innovation, Public Health Service of Amsterdam, Amsterdam, The Netherlands

**Keywords:** SARS-CoV-2, COVID-19, Hospitalisation, Ethnicity, Geographical, Socio-economic status

## Abstract

**Background:**

It is important to gain insight into the burden of COVID-19 at city district level to develop targeted prevention strategies. We examined COVID-19 related hospitalisations by city district and migration background in the municipality of Amsterdam, the Netherlands.

**Methods:**

We used surveillance data on all PCR-confirmed SARS-CoV-2 hospitalisations in Amsterdam until 31 May 2020, matched to municipal registration data on migration background. We calculated directly standardised (age, sex) rates (DSR) of hospitalisations, as a proxy of COVID-19 burden, per 100,000 population by city district and migration background. We calculated standardised rate differences (RD) and rate ratios (RR) to compare hospitalisations between city districts of varying socio-economic and health status and between migration backgrounds. We evaluated the effects of city district and migration background on hospitalisation after adjusting for age and sex using Poisson regression.

**Results:**

Between 29 February and 31 May 2020, 2326 cases (median age 57 years [IQR = 37–74]) were notified in Amsterdam, of which 596 (25.6%) hospitalisations and 287 (12.3%) deaths. 526/596 (88.2%) hospitalisations could be matched to the registration database. DSR were higher in individuals living in peripheral (South-East/New-West/North) city districts with lower economic and health status, compared to central districts (Centre/West/South/East) (RD = 36.87,95%CI = 25.79–47.96;RR = 1.82,95%CI = 1.65–1.99), and among individuals with a non-Western migration background compared to ethnic-Dutch individuals (RD = 57.05,95%CI = 43.34–70.75; RR = 2.36,95%CI = 2.17–2.54). City district and migration background were independently associated with hospitalisation.

**Conclusion:**

City districts with lower economic and health status and those with a non-Western migration background had the highest burden of COVID-19 during the first wave of COVID-19 in Amsterdam.

**Supplementary Information:**

The online version contains supplementary material available at 10.1186/s12889-021-11782-w.

## Introduction

The first cases of coronavirus disease 2019 (COVID-19) were reported at the end of 2019 in Wuhan, China. Severe acute respiratory syndrome coronavirus 2 (SARS-CoV-2), the virus causing COVID-19, has since rapidly spread across the globe. The World Health Organization (WHO) consequently declared COVID-19 a global epidemic on 11 March 2020. By the end of June 2021, over 180 million cases and almost 4 million COVID-19 related deaths had been reported worldwide [1].

In the Netherlands, COVID-19 was declared a Group A notifiable disease under the Public Health legal act on 28th January 2020 [2]. This required clinicians and laboratories to immediately notify the regional public health service (PHS) every suspected and/or confirmed case of COVID-19. The first confirmed case of COVID-19 on 27 February 2020 had an epidemiological link to Northern Italy. Initial sporadic clusters led to a subsequent nationwide spread of the virus, including in the capital city of Amsterdam. From 12 to 15 March, the Dutch government initiated a series of restrictions [3] which were gradually lifted from 11 May onwards. Up to 1 June 2021, the Netherlands had reported approximately 46,000 confirmed cases and over 6000 COVID-19 deaths [4].

The regional PHS of Amsterdam acts upon notifications of infectious diseases including COVID-19 for the wider Amsterdam-Amstelland area, consisting of 6 municipalities including Amsterdam, with a total regional population of about 1,06 million. Anecdotal reports from hospital staff in April 2020 suggested that a disproportionate number of patients of ethnic minority background had been admitted to hospitals and intensive care units (ICU) in Amsterdam. This echoed reports from the United Kingdom [5] (UK) and United States of America [6] (USA) at the time, which suggested that age- and sex-standardised mortality rates were highest among socio-economically deprived groups and ethnic minority groups; findings that were later supported by a systematic review [7]. Early analyses also demonstrated that individuals with a migration background in the Netherlands had higher rates of excess mortality (i.e. higher than would be expected in ‘normal’ conditions) compared to individuals of Dutch origin, in particular in those with a non-Western migration background [8]. Amsterdam is an increasingly ethnically diverse city with more than half the population having a migration background [9]. Disparities in the distribution of communicable and non-communicable diseases have previously been demonstrated in the city between individuals with and without a migration background [10], even when matched for age- and socio-economic status (SES) [11]. In addition, health inequalities have been reported between the peripheral city districts of Amsterdam with lower average income [12] and the central, higher-income city districts of the Centre, West and East districts, with poorer health outcomes being reported in peripheral districts [13]. Investigating whether such disparities also exist for COVID-19 burden is crucial to developing targeted prevention strategies.

We therefore aimed to examine differences in burden of COVID-19 hospitalisations between city districts and ethnic groups in the city of Amsterdam, using routinely collected surveillance data from 29 February to 31 May 2020.

## Methods

### COVID-19 case definition and test strategy

Up to and including 11th March 2020, SARS-CoV-2 testing in the Netherlands was conducted only in individuals who fulfilled a strict case definition: (i) having an epidemiological link to a confirmed case and/or returning from a high-risk region with widespread transmission within 14 days prior to the onset of symptoms, and (ii) the presence of fever with at least one of the following symptoms: coughing, shortness of breath (dyspnoea). Testing was mainly carried out by a home testing team of the PHS, by general practitioners and in hospitals.

After restrictions were initiated between 12 and 15 March 2020, testing no longer required a confirmed epidemiological link or travel history, but instead focussed on mitigating the impact on frontline healthcare services and protecting vulnerable groups. Healthcare workers, residents of long-term care facilities and individuals at high risk of severe disease with COVID-like symptoms were prioritised. For patients, testing was carried out in the hospital setting (including purpose-built COVID-19 triage and testing tents), by family physicians, and by the PHS’s home testing team, who also performed testing in long-term care facilities. Healthcare workers could be tested at a special test location at the PHS site itself or at work (in hospitals) and were prioritised in situations where a staffing shortage was at stake.

From 11 May 2020, testing at the PHS site was also made available to teachers and those working in other contact professions with COVID-like symptoms but remained inaccessible for the general public until 1 June 2020. Because of this restrictive test policy during the period analysed, we used hospitalisations as a marker of epidemic progression.

### Data collection, source and linkage

Since 28th January 2020, all persons with a positive PCR test for SARS-CoV-2 are required to be notified to the regional PHS, which subsequently notifies the National Institute for Public Health and the Environment (RIVM). In the wider region of Amsterdam-Amstelland, all positive cases are notified to the regional PHS of Amsterdam. For this study, we retrieved secondary data collected between 29 February (date of first confirmed case) and 31 May 2020 from the Amsterdam-Amstelland COVID-19 notification database, the surveillance database by the PHS of Amsterdam on all notified cases, hospitalisations and deaths residing in the municipality of Amsterdam. These data included information on age and sex of the case, and whether the case had worked as a health care worker or was a resident in a long-term care facility. We matched surveillance data to registration data from the municipality records of the City of Amsterdam (BRP) to retrieve postal code and the country of birth of the case and their parents. Since this study made use of routine surveillance data, no human participants were involved. The database is not publicly available.

### Determination of city district and migration background

We determined city district based on postal code of current residence [14]. We assessed migration background and generation based on the country of birth of individuals and their parents [15]. Dutch ethnic origin, i.e. having no migration background, was defined as having parents who were both born in the Netherlands. An individual was considered of non-Dutch ethnicity if he/she was born abroad and had at least one parent who was born abroad (first generation) or he/she was born in the Netherlands and both parents were born abroad (second generation) [16]. For second generation migrants of whom both parents were born abroad, the mother’s country of birth was leading in defining migration background. Individuals of non-Dutch ethnicity were further classified into having a non-Western migration background (from African, Latin-American or Asian countries, or Turkey, excluding Indonesia and Japan) or Western migration background (from North-American, European or Oceanian countries, or Indonesia or Japan, excluding Turkey) according to the definition of the Dutch Central Bureau of Statistics [16].

### Statistical analysis

We evaluated the number of cases, hospitalisations and deaths, overall and over time (notification date and symptom onset). In the epidemiological curve by self-reported date of symptom onset, missing dates were imputed, assuming that all those who were tested had symptoms. First, we created a distribution of time between symptom onset and case notification for those with a known date of symptom onset. Second, we randomly sampled from this distribution to estimate the date of symptom onset if this was missing.

We calculated crude and directly standardised rates (DSR) of hospitalisations per 100,000 population, overall and by city district and migration background. Rates were standardised for age (≤14, 15–29, 29–44, 45–59, 60–74, ≥75 years) and sex (female, male). 95% confidence intervals (CI) were calculated using the gamma method [17, 18]. We computed rate differences (RD) and rate ratios (RR) to compare DSR of hospitalisation between (i) city districts, (ii) migration backgrounds (first and second generation migrants combined) and (iii) a combination of both variables, i.e. six strata of city districts (dichotomized into the central districts with higher average incomes (Central/West/South/East) and peripheral districts with lower average incomes (Southeast/North/New-West), based on 2018 income per capita per city district [12]) and migration background (none [ethnic-Dutch], Western, non-Western). First and second generation were combined as this was highly correlated with age and a stratified analysis would result in few outcomes in specific age-stratums. Since elderly persons with a migration background are less likely to engage with home care or nursing homes [19], we hypothesized that they would be more likely to be hospitalised upon a deterioration in health than ethnic-Dutch elderly, who may be more likely to receive palliative care in the community care setting. To explore this possible bias, we conducted an additional analysis of DSR of hospitalisation by migration background, stratified by age < 60 and ≥ 60 years.

We evaluated the individual effects of city district (peripheral, central), migration background, sex and age in a Poisson regression model, using the log of the population size per district/background/sex/age stratum as an offset. In this model, we sequentially added interaction terms between migration background and (1) city district, (2) age, and (3) sex which were tested for significance using likelihood ratio tests.

We assumed statistical significance at a *P*-value< 0·05. We used the dsr package in R [18] to calculate DSR, RD and RR. All analyses were performed in R (version 3.6.3, Vienna, Austria).

## Results

**Fig. 1:**
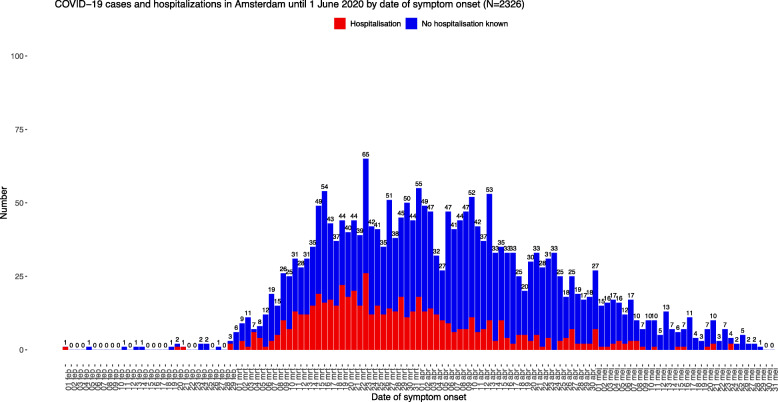
COVID-19 notifications in Amsterdam between 29 February and 31 May 2020

Between 29 February 2020 and 31 May 2020, 2326 COVID-19 cases were notified in Amsterdam, of which 596 (25.6%) hospitalisations and 287 (12.3%) deaths. The number of new cases peaked mid-March, shown by date of symptom onset in Fig. 1 and by notification date in Supplementary Figure S1. Characteristics of COVID-19 cases by hospitalisation status are presented in Table 1. Median age of hospitalised cases was higher (64 [IQR 51–73]) than cases for which hospitalisation status was non-hospitalised or unknown (54 [IQR 34–74]). The majority of hospitalised cases were male (361/596 [60.6%]), while most cases overall were female (1346 [57.9%]). 814 (35.0%) of all notifications were registered as health care workers, of whom 314 (38.6%) worked in a long-term care facility.

**Table 1:** Characteristics of COVID-19 cases and hospitalisations in Amsterdam, the Netherlands, 29 February-31 May 2020

	Total (***N*** = 2326)		Hospitalised (***n*** = 596)		Not hospitalised/ unknown (***n*** = 1730)	
Characteristic	n	%	n	%	n	%
**Age in years, median [IQR]**	57	[37–74]	64	[51–73]	54	[34–74]
**Sex**						
Female	1346	57.9	230	38.6	1116	64.5
Male	965	41.5	361	60.6	604	34.9
Unknown	15	0.6	5	0.8	10	0.6
**Health care worker (HCW)**						
No	1108	47.6	383	64.3	725	41.9
Yes	814	35.0	47	7.9	767	44.3
HCW not in a long-term care facility/unknown location	500	61.4	36	76.6	464	60.5
HCW in a long-term care facility	314	38.6	11	23.4	303	39.5
Unknown	404	17.3	166	27.8	238	13.8
**Resident of a long-term care facility**						
No/unknown	1867	80.3	579	97.2	1299	74.5
Yes	459	19.7	17	2.9	445	25.5
**City district**						
Centre	162	7.0	37	6.2	141	7.0
New-West	433	18.6	130	21.8	360	18.0
North	268	11.5	82	13.8	239	12.0
East	377	16.2	62	10.4	330	16.5
West	341	14.7	86	14.4	293	14.6
South	350	15.1	77	12.9	312	15.6
South-East	355	15.3	113	19.0	320	16.0
Unknown	40	1.7	9	1.5	7	0.4
**Died**						
No/unknown	2039	87.7	496	83.2	1543	89.2
Yes	287	12.3	100	16.8	187	10.8

*Abbreviations*: *HCW* health care worker, *IQR* interquartile range

Of the 2326 cases, 2002 (86.1%) could be matched to the registration database, including 526/596 hospitalisations (88.2%) and 235/287 (81.8%) deaths (Supplementary Figure S2, Supplementary Table S1). Table 2 shows hospitalisation DSR per 100,000 population by city district and migration background in the matched population. Hospitalisation DSR were almost two-fold higher in individuals living in peripheral city districts compared to central districts (*RR* = 1.82, 95%CI = 1.65–1.99), with 36.87 additional hospitalisations per 100.000 population (RD = 36.87, 95%CI = 25.79–47.96). The difference between peripheral and central city districts became increasingly apparent over time (Figure S3). Further stratification by individual city district is presented in Supplementary Table S2.

**Table 2:** Hospitalisation rates by city district and migration among those linked to the registration database

	Hospital admissions	Population ^**a**^	Crude rate per 100,000 population (95% CI)	Standardised rate per 100,000 population ^**b**^ (95% CI)	Standardised rate difference (95% CI)	Standardised rate ratio (95% CI)
**Total**^**c**^	523	873,055	59.90 (54.88–65.27)			
**City district**						
Central (C/W/S/E)	233	523,536	44.51 (38.97–50.6)	44.99 (39.40–51.16)	Ref.	Ref.
Peripheral (SE/N/NW)	289	349,519	82.69 (73.43–92.79)	81.87 (72.68–91.89)	36.87 (25.79–47.96)	1.82 (1.65–1.99)
**Migration background**						
None (ethnic-Dutch)	204	386,521	52.78 (45.78–60.54)	42.04 (36.38–48.32)	Ref.	Ref.
Non- Western	262	316,720	82.72 (73.01–93.37)	99.08 (87.08–112.28)	57.05 (43.34–70.75)	2.36 (2.17–2.54)
Western	57	169,814	33.57 (25.42–43.49)	41.06 (30.96–53.4)	−0.98 (−13.28–11.32)	0.98 (0.68–1.27)

^a^ Population on 1 April 2020

^b^ Standardised for age (in 15-year groups) and gender, using the total population of Amsterdam as the standard population

^c^ 3 matched cases had missing data

*Abbreviations: C* Centre, *CI* confidence interval, *E* East, *N* North, *NW* North-West, *SE* South-East, *W* West

Hospitalisation DSR were more than two-fold higher in individuals with a non-Western migration background compared to ethnic-Dutch individuals (*RR* = 2.36, 95%CI = 2.17–2.54), with 57 additional hospitalisations per 100.000 population (RD = 57.05, 95%CI = 43.34–70.75). No statistically significant difference was observed between individuals with a Western migration background and ethnic-Dutch individuals. Further stratification by specific large non-Western ethnic groups living in Amsterdam (Dutch Antillean, Moroccan, Surinamese, Turkish and Ghanaian) is presented in Supplementary Table S3. Compared to the ethnic-Dutch population, the hospitalisation RR were highest among individuals of Ghanaian (*RR* = 4.25, 95%CI = 3.31–5.19; RD = 136.75, 95%CI = -29.83–303.33) and Turkish ethnic origin (*RR* = 3.08, 95%CI = 2.72–3.43; RD = 87.31, 95%CI = 44.51–130.11). In our additional age-stratified analysis, we found that individuals < 60 years with a non-Western migration background had a three-fold higher DSR compared to ethnic-Dutch individuals < 60 years (RR = 3.18, 95%CI = 2.85–3.52), while the DSR was reduced but remained almost two-fold higher in individuals ≥60 years (RR = 1.83, 95% CI: 1.57–2.09) (Supplementary Tables S4 and S5).

**Fig. 2:**
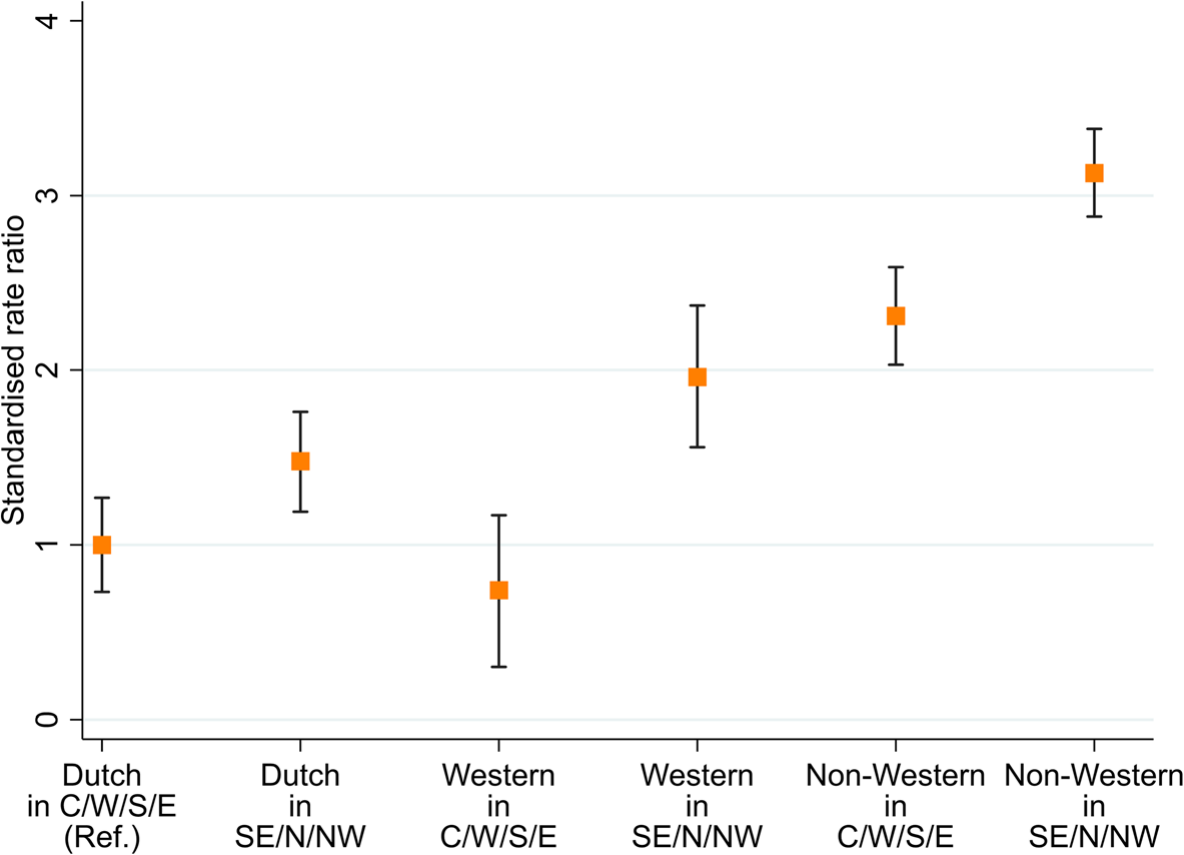
Standardised rate ratios comparing hospitalisation rates between migration backgrounds and city districts in Amsterdam

After stratifying by city district, the DSR was highest in non-Western residents of peripheral districts (Supplementary Figure S4): three times greater than in ethnic-Dutch residents of central districts *RR* = 3.13, 95%CI = 2.88–3.38; RD = 76.64, 95%CI = 57.38–95.89) (Fig. 2).

In multivariable Poisson regression, non-Western migration background, living in a peripheral city district, male sex and older age were associated with hospitalisation (Table 3). No statistically significant interactions were found between migration background and city district (*P =* 0.08, Supplementary Table S6), migration background and age (*P =* 0.33, Supplementary Table S7) and migration background and sex (*P* = 0.45, Supplementary Table S8).

**Table 3:** Characteristics associated with COVID-19 related hospitalization

Characteristic	aRR (95% CI)	***P***-value
**Migration background**		
None (ethnic-Dutch)	1	
Western	0.97 (0.71–1.29)	0.83
Non-Western	2.20 (1.82–2.67)	< 0.001
**City district**		
Central (C/W/S/E)	1	
Peripheral (SE/N/NW)	1.53 (1.28–1.82)	< 0.001
**Sex**		
Male	1	
Female	0.59 (0.49–0.70)	< 0.001
**Age**		
< 45 years	1	
45–59 years	4.95 (3.73–6.6)	< 0.001
60–74 years	13.52 (10.47–17.63)	< 0.001
≥ 75 years	23.84 (17.92–31.88)	< 0.001

*Abbreviations*: *aRR* adjusted rate ratio, *C* Centre, *CI* confidence interval, *E* East, *N* North, *NW* North-West, *SE* South-East, *W* West

Estimates were obtained from a multivariable Poisson regression model

## Discussion

We identified geographical and ethnic disparities in the burden of COVID-19 in Amsterdam during the first wave of COVID-19. Specifically, people living in peripheral, lower-income city districts (New-West, North or South-East) with a non-Western migration background had three-fold higher hospitalisation rates compared to ethnic-Dutch individuals living central, higher-income city districts (Centre, West, South or East).

Previous research has demonstrated similar health disparities between city districts in Amsterdam [13]. A quadrennially repeating comprehensive health survey has consistently shown that residents of peripheral city districts are more likely to suffer from comorbidities and be overweight or obese than residents of central districts [13]. As chronic lifestyle conditions such as type 2 diabetes mellitus and cardio-vascular disease have been shown to increase the risk of developing severe COVID-19 symptoms [20], this suggests that the higher rate of COVID-19 hospitalisations seen in these city districts might be partially explained by a higher prevalence of chronic comorbidities. In addition, the peripheral districts have been shown to have a higher percentage of vulnerable residents [13], based on a vulnerability score that encompasses income, job security and healthcare expenditure. The interplay between SES and risk of both SARS-CoV-2 infection and severe disease has been previously documented [21]. For example, individuals with a lower SES may be more likely to work as essential frontline workers – including healthcare workers and those working in sectors such as cleaning, construction, and transport [22] – with a reduced opportunity to work from home [23]. Lower SES may also lead to a greater reliance on public transport, larger multi-generational households and smaller house size per person [24], all of which may increase the risk of exposure to infection [22, 25]. We therefore postulate that the greater burden of COVID-19 hospitalisation seen in these districts is due to a complex interaction between different factors. Indeed, when restricting to individuals < 60 years of age in our analyses, the hospitalisation RR in those with a non-Western migration background compared to the ethnic-Dutch group remained and was even accentuated. This suggests that the differences in hospitalisation rates cannot be fully explained by an increased tendency of non-Western migrants, compared to ethnic-Dutch elderly, to be treated in hospital instead of receiving palliative care in the community setting or in nursing homes 26. The higher RR in younger age groups might imply that increased exposure secondary to having a public-facing occupation or higher levels of other causes of increased exposure among those with a non-Western migration background may have played an important role. Further elucidating the causal factors underlying SES disparities in COVID-19 burden requires further research.

In addition, our analysis demonstrated that individuals with a non-Western migration background, including groups with a Moroccan, Turkish, Surinamese and Ghanaian background, had a higher COVID-19 burden, even when stratifying by city district. This is in line with previous findings from the UK and USA and, more recently, other high-income countries such as Norway [27]. In the UK, the risk of death among those with a COVID-19 diagnosis was twice as high for people of Bangladeshi ethnicity compared to people of White ethnicity, after adjusting for sex, age, deprivation and region [21]. Additional studies demonstrated that, even when adjusting for age, sex, comorbidities and several SES-related determinants (though notably not public-facing occupation), hospitalisation and ICU admission rates among Black and Asian Minority Ethnic individuals were higher compared to ethnically White individuals [28], suggesting that ethnicity plays an independent role in explaining these disparities. In the US, age-adjusted hospitalisation rates were 5.3 and 4.7 times higher in American Indian or Alaska Native and Black or African American persons respectively, compared to non-Hispanic white persons; although this analysis was not adjusted for social inequalities [6]. Whilst these findings consistently demonstrate that the burden of COVID-19 varies by ethnicity, the contribution of underlying cultural norms, health literacy, and differences in health-seeking behaviour has yet to be revealed.

Considering our findings substantiate reports of increased COVID-19 burden among ethnic minority groups in other countries, it is concerning that information on ethnicity is often not collected in routine surveillance systems in many countries [29]. This results in these important differences in disease burden being concealed within the data, hindering the ability to set up targeted initiatives to reach particularly vulnerable populations. As stated by Tai et al. [30], further research on the interplay between inherent social inequalities and ethnicity is clearly required to ensure optimal surveillance of the impact of COVID-19 on vulnerable groups, to disentangle whether the increased burden is the result of a higher infection rate, the severity of disease manifestation, or both, and to inform and develop appropriate and accessible prevention strategies. Indeed, local prevention teams have now been deployed in Amsterdam to help reduce ethnic inequalities in COVID-19 burden, using strategies such as mobile test buses in neighbourhoods with a high rate of diagnosed COVID-19 infections per population and information provision on preventive measures tailored to specific groups.

An important limitation of our study is that the surveillance data paint an incomplete picture of the first wave of the outbreak, as cases were underreported due to selective testing and data collection was limited. We used the hospitalisation rate per 100,000 population as a marker of outbreak progression, but this limits the distinction that can be made between risk of infection and risk of severe disease requiring hospital admission. In addition, hospitalisations and deaths among already notified cases may also have been underreported, and we were unable to match all notifications to the municipal register. Furthermore, absence of key individual socio-demographic, socio-economic, and clinical characteristics limits the inferences that can be made about causal factors on an individual patient level. For example, we used city district as an imperfect proxy for SES, but SES at the community or individual level within each city district may have been different. By further stratifying groups by migration background and complementing this with qualitative research (for example, through community focus groups) more insight can be gained into which community-specific targeted prevention strategies may help minimise the disproportionate distribution of COVID-19 in Amsterdam.

Our study is the first in the Netherlands to link surveillance data with registration data on migration background to demonstrate the unequal distribution of the burden of COVID-19 within the city of Amsterdam. We show that substantial differences in COVID-19 hospitalisation rates exist between city districts and ethnic groups in Amsterdam. Our findings corroborate reports from other high-income countries to suggest that public health efforts worldwide must be focussed on mitigating further impact of COVID-19 upon communities at highest risk of both infection and serious disease. Action must be taken to strengthen targeted prevention strategies which address the needs of affected communities.

## Supplementary Information

**Additional file 1:.** Supplementary materials contain Supplementary Figures 1 to 4 and Supplementary Tables 1 to 8.

## Data Availability

Information on can be obtained from the corresponding author, Liza Coyer, lcoyer@ggd.amsterdam.nl.

## References

[1] Dong E, Du H, Gardner L (2020). An interactive web-based dashboard to track COVID-19 in real time. Lancet Infect Dis.

[2] COVID-19 | LCI richtlijnen. [cited 2020 Oct 17]. Available from: https://lci.rivm.nl/richtlijnen/covid-19

[3] Ministerie van Onderwijs C en W, Veiligheid M, Ministerie van Volksgezondheid W en S. Nieuwe maatregelen tegen verspreiding coronavirus in Nederland - Nieuwsbericht - Rijksoverheid.nl: Ministerie van Algemene Zaken; 2020. [cited 2020 Dec 3]. Available from: https://www.rijksoverheid.nl/actueel/nieuws/2020/03/12/nieuwe-maatregelen-tegen-verspreiding-coronavirus-in-nederland

[4] Coronavirus Disease (COVID-19) Situation Reports. Available from: https://www.who.int/emergencies/diseases/novel-coronavirus-2019/situation-reports. Accessed 30 June 2021.

[5] Pareek M, Bangash MN, Pareek N, Pan D, Sze S, Minhas JS (2020). Ethnicity and COVID-19: an urgent public health research priority. Lancet.

[6] CDC. Coronavirus Disease 2019 (COVID-19): Centers for Disease Control and Prevention; 2020. Available from: https://www.cdc.gov/coronavirus/2019-ncov/covid-data/investigations-discovery/hospitalization-death-by-race-ethnicity.html. Accessed 3 Dec 2020.

[7] Sze S, Pan D, Nevill CR, Gray LJ, Martin CA, Nazareth J, et al. Ethnicity and clinical outcomes in COVID-19: A systematic review and meta-analysis. EClinicalMedicine. 2020;29. Available from: https://www.thelancet.com/journals/eclinm/article/PIIS2589-5370(20)30374-6/abstract. Accessed 13 Jan 2021. 10.1016/j.eclinm.2020.100630 PMC7658622 33200120

[8] Kunst A, de Visser M, Stoeldraijer L, Harmsen C. Oversterfte tijdens de eerste zes weken van de corona-epidemie: Sociaal-demografische en geografische verschillen: CBS; 2020. Available from: https://www.cbs.nl/-/media/_pdf/2020/20/oversterfte-tijdens-de-coronaepidemie.pdf

[9] Statistiek CB voor de. Hoeveel mensen met een migratieachtergrond wonen in Nederland?. Centraal Bureau voor de Statistiek. Available from: https://www.cbs.nl/nl-nl/dossier/dossier-asiel-migratie-en-integratie/hoeveel-mensen-met-een-migratieachtergrond-wonen-in-nederland. Accessed 15 Jan 2021.

[10] Urbanus AT, van de Laar TJ, van den Hoek A (2011). Hepatitis C in the general population of various ethnic origins living in the Netherlands: should non-Western migrants be screened?. J Hepatol.

[11] Ujcic-Voortman JK, Schram MT, Jacobs-van der Bruggen MA, Verhoeff AP, Baan CA (2009). Diabetes prevalence and risk factors among ethnic minorities. Eur J Pub Health.

[12] Centraal Bureau voor de Statistiek (CBS). Gemiddeld persoonlijk inkomen (incl. studenten) naar stadsdelen, 2014–2018. Gemeente; 2020. Available from: https://api.data.amsterdam.nl/dcatd/datasets/zxlGIg_ibtjfPg/purls/1. Accessed 15 Jan 2021.

[13] Amsterdam. Gezondheid in Beeld. GGD Amsterdam. Gemeente Amsterdam; [cited 2020 Dec 15]. Available from: https://www.ggd.amsterdam.nl/beleid-onderzoek/gezondheidsmonitors/gezondheid-beeld-0/. Accessed 15 Dec 2020.

[14] Amsterdam. Districts and neighbourhoods. English site. Gemeente Amsterdam; Available from: https://www.amsterdam.nl/en/districts/. Accessed 13 Dec 2020.

[15] Stronks K, Kulu-Glasgow I, Agyemang C. The utility of “country of birth” for the classification of ethnic groups in health research: the Dutch experience. Ethnicity Health. 2009;14. Available from: https://pubmed.ncbi.nlm.nih.gov/19052941/. Accessed 3 Dec 2020. 10.1080/13557850802509206 19052941

[16] Statistiek CB voor de. Migratieachtergrond. Centraal Bureau voor de Statistiek. Available from: https://www.cbs.nl/nl-nl/onze-diensten/methoden/begrippen/migratieachtergrond. Accessed 3 Dec 2020.

[17] Fay MP, Feuer EJ (1997). Confidence intervals for directly standardized rates: a method based on the gamma distribution. Stat Med.

[18] dsr package | R Documentation. Available from: https://www.rdocumentation.org/packages/dsr/versions/0.2.2. Accessed 3 Dec 2021.

[19] Denktaş S, Koopmans G, Birnie E, Foets M, Bonsel G (2009). Ethnic background and differences in health care use: a national cross-sectional study of native Dutch and immigrant elderly in the Netherlands. Int J Equity Health.

[20] Yang J, Zheng Y, Gou X, Pu K, Chen Z, Guo Q, Ji R, Wang H, Wang Y, Zhou Y (2020). Prevalence of comorbidities and its effects in patients infected with SARS-CoV-2: a systematic review and meta-analysis. Int J Infect Dis.

[21] Public Health England (2020). Disparities in the risk and outcomes of COVID-19.

[22] Identifying Critical Infrastructure During COVID-19 | CISA. Available from: https://www.cisa.gov/identifying-critical-infrastructure-during-covid-19. Accessed 3 Dec 2020.

[23] Statistiek CB voor de. Beroep naar migratieachtergrond: Centraal Bureau voor de Statistiek; 2019. [cited 2020 Dec 3]. Available from: https://www.cbs.nl/nl-nl/maatwerk/2020/26/beroep-naar-migratieachtergrond-2019. Accessed 3 Dec 2020.

[24] Huishoudens naar migratieachtergrond. [cited 2020 Dec 3]. Available from: https://www.cbs.nl/nl-nl/cijfers/detail/70067ned. Accessed 3 Dec 2020.

[25] Blau FD, Koebe J, Meyerhofer PA (2020). Who are the Essential and Frontline Workers?.

[26] Suurmond J, Rosenmöller DL, El Mesbahi H, Lamkaddem M, Essink-Bot M-L (2016). Barriers in access to home care services among ethnic minority and Dutch elderly--a qualitative study. Int J Nurs Stud.

[27] Indseth T, Grøsland M, Arnesen T, Skyrud K, Kløvstad H, Lamprini V, Telle K, Kjøllesdal M (2021). COVID-19 among immigrants in Norway, notified infections, related hospitalizations and associated mortality: a register-based study. Scand J Public Health.

[28] Lassale C, Gaye B, Hamer M, Gale CR, Batty GD. Ethnic disparities in hospitalization for COVID-19: a community-based cohort study in the UK. MedRxiv. 2020;88:44–9. 10.1016/j.bbi.2020.05.074 PMC7263214 32497776

[29] Labgold K, Hamid S, Shah S, Gandhi NR, Chamberlain A, Khan F, et al. Measuring the missing: greater racial and ethnic disparities in COVID-19 burden after accounting for missing race/ethnicity data. MedRxiv. 2020. 10.1101/2020.09.30.20203315. 10.1097/EDE.0000000000001314 PMC8641438 33323745

[30] Tai DB, Shah A, Doubeni CA, Sia IG, Wieland ML. The Disproportionate Impact of COVID-19 on Racial and Ethnic Minorities in the United States. Clin Infect Dis. 2020; Available from: https://www.ncbi.nlm.nih.gov/pmc/articles/PMC7337626/. Accessed 16 Oct 2020. 10.1093/cid/ciaa815 PMC7337626 32562416

