# Supplementary material for: Hospitalisation rates differed by city district and ethnicity during the first wave of COVID-19 in Amsterdam, The Netherlands
Source: BMC Public Health. 2021 Sep 22;21:1721. doi: 10.1186/s12889-021-11782-w (PMC8456400; doi:10.1186/s12889-021-11782-w)
Supplement: Supplementary file 1 — Additional file 1:. Supplementary materials contain Supplementary Figures 1 to 4 and Supplementary Tables 1 to 8. [file 12889_2021_11782_MOESM1_ESM.docx]

# Supplement to: Hospitalisation rates differed by city district and ethnicity during the first wave of COVID-19 in Amsterdam, the Netherlands

Liza Coyer*, Elke Wynberg1*, Marcel Buster, Camiel Wijffels, Maria Prins, Anja Schreijer, Yvonne T.H.P van Duijnhoven, Alje van Dam, Mariken van der Lubben, Tjalling Leenstra

*Contributed equally

**Content Page**

**Figures**

Figure S1. COVID-19 notifications from 29 February until 31 May 2020 **2**
by notification date, stratified by hospitalisation status, Amsterdam, the Netherlands

Figure S2. Flowchart depicting COVID-19 cases notified in the municipality of Amsterdam **3**

between 29 February and 31 May 2020, and linkage of notification data with municipality registration data, the Netherlands

Figure S3. Cumulative hospitalisation rate per 100,000 population in Amsterdam over week of **4**

symptom onset between 29 February and 31 May 2020 among those matched with the
municipal registration database, by binary representation of city districts

Figure S4. Crude and standardised hospitalisation rates per 100,000 population by migration **5** background and city district in the municipality of Amsterdam between 29 February and
31 May 2020

**Tables**

Table S1. Characteristics of COVID-19 cases who could and could not be matched to the **6**
municipality registration database in Amsterdam, the Netherlands, 29 February to 31 May 2020

Table S2. Hospitalisation rates by city district among those linked to the **7**
registration database, Amsterdam, the Netherlands, 29 February to 31 May 2020

Table S3. Hospitalisation rates by migration background (first and second generation **8**
combined) among those linked to the registration database, Amsterdam, the Netherlands,
29 February to 31 May 2020

Table S4. Hospitalisation rates by migration history (first and second generation combined) **9**

among those aged <60 years linked to the registration database, Amsterdam, the Netherlands,
29 February to 31 May 2020

Table S5. Hospitalisation rates by migration history (first and second generation combined) **10**

Among those aged ≥60 years linked to the registration database, Amsterdam, the Netherlands,
29 February to 31 May 2020

Table S6. Associations with COVID-19 related hospitalisation, obtained from a Poisson model **11**
with an interaction term between migration background and city district, Amsterdam,
the Netherlands, 29 February to 31 May 2020

Table S7. Associations with COVID-19 related hospitalisation, obtained from a Poisson model **12**
with an interaction term between migration background and city district, Amsterdam,
the Netherlands, 29 February to 31 May 2020

Table S8. Associations with COVID-19 related hospitalisation, obtained from a Poisson model **13**
with an interaction term between migration background and city district, Amsterdam,
the Netherlands, 29 February to 31 May 2020

**Figure S1. COVID-19 notifications from 29 February until 31 May 2020 by notification date, stratified by hospitalisation status, Amsterdam, the Netherlands**


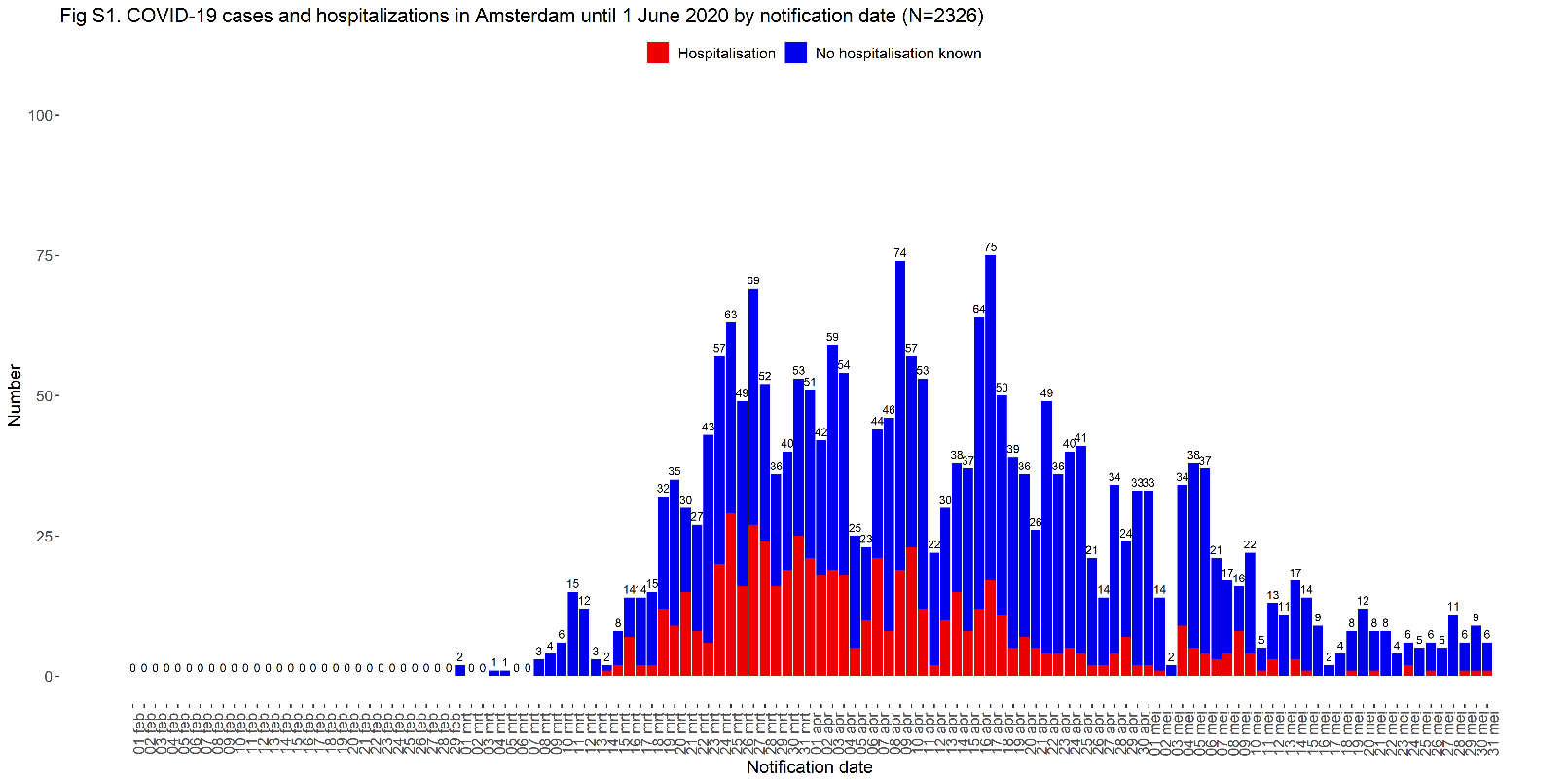


**Figure S2. Flowchart depicting COVID-19 cases notified in the municipality of Amsterdam between 29 February and 31 May 2020, and linkage of notification data with municipality registration data, the Netherlands**

2867 cases notified in Amsterdam – Amstelland between 29 February and 31 May 2020

519 not living in municipality of Amsterdam:

301 Amstelveen

100 Diemen

48 Uithoorn

38 Aalsmeer

32 Ouder-Amstel

13 unknown municipality

9 passers-by

2326 cases living in municipality of Amsterdam:
 596 (25.6%) hospitalisations
 287 (12.3%) deaths

2002 matched with registration database:

526 (26.3%) hospitalisations

235 (11.7%) deaths

2 no match with registration database:

0 hospitalisations

1 death

322 (13.8%) no citizen service number available at time of data linkage

70 (21.7%) hospitalisations

51 (15.8%) deaths

2004 (86.2%) citizen service number available


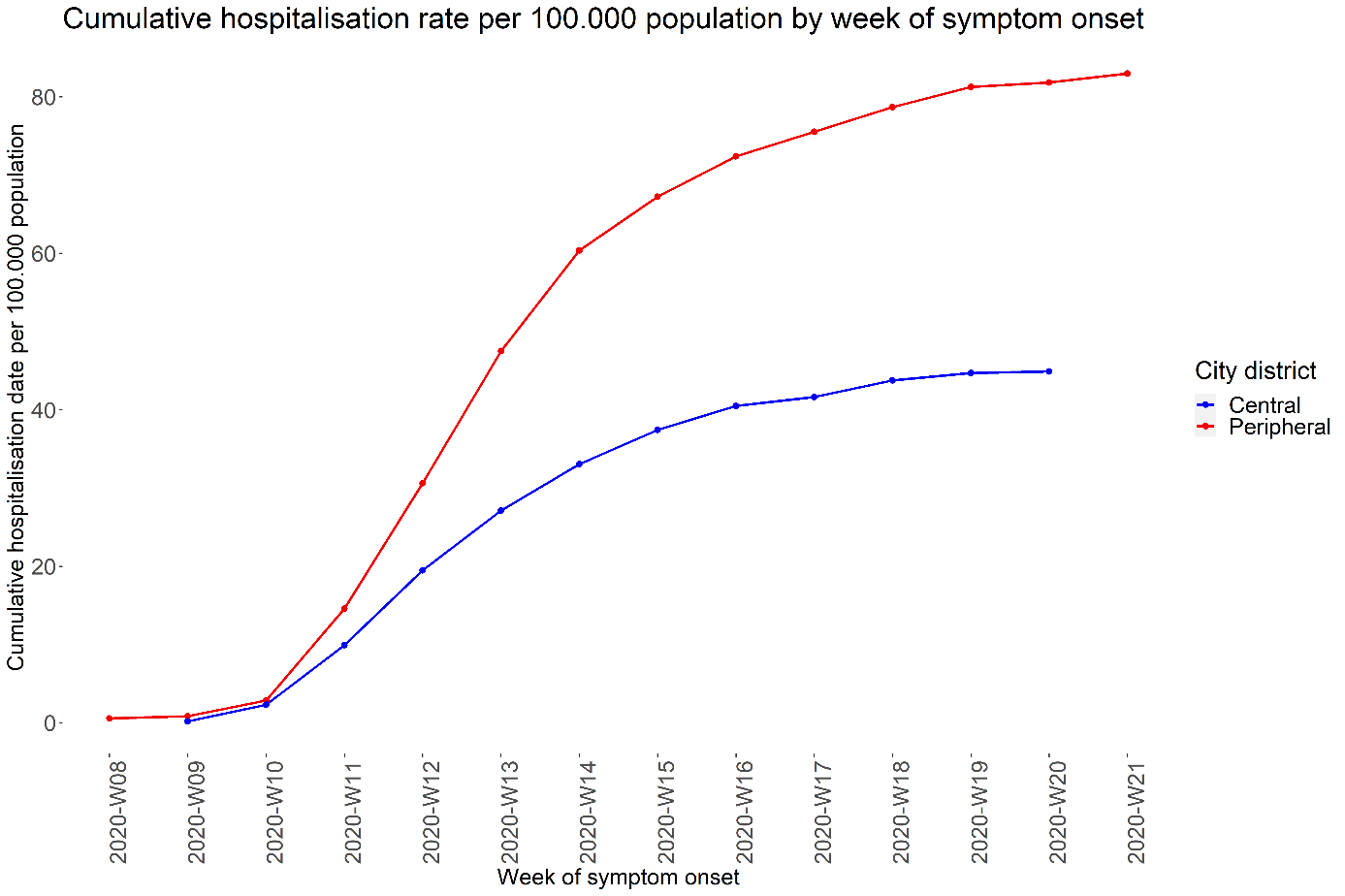
**Figure S3. Cumulative hospitalisation rate per 100,000 population in Amsterdam over week of symptom onset between 29 February and 31 May 2020 among those matched with the municipal registration database, by binary representation of city districts**

**Figure S4: Crude and standardised hospitalisation rates per 100,000 population by migration background and city district in the municipality of Amsterdam between 29 February and 31 May 2020**

**Table S1. Characteristics of COVID-19 cases who could and could not be matched to the municipality registration database in Amsterdam, the Netherlands, 29 February to 31 May 2020**

|  | **Total**  **(N=2326)** | | **Matched**  **(n=2002)** | | **Not matched**  **(n=324)** | |
| --- | --- | --- | --- | --- | --- | --- |
| **Characteristic** | **n** | **%** | **n** | **%** | **n** | **%** |
| **Age in years, median [IQR]** | 57 | [37-74] | 56 | [37-73] | 62 | [44-80] |
| **Sex** |  |  |  |  |  |  |
| Female | 1346 | 57.9 | 1181 | 59.0 | 165 | 50.9 |
| Male | 965 | 41.5 | 809 | 40.4 | 156 | 48.2 |
| Unknown | 15 | 0.6 | 12 | 0.6 | 3 | 0.9 |
| **Health care worker (HCW)** |  |  |  |  |  |  |
| No | 1108 | 47.6 | 934 | 46.7 | 174 | 53.7 |
| Yes | 814 | 35.0 | 739 | 46.7 | 75 | 23.2 |
| HCW not in a long-term care  facility/unknown location | 500 | 61.4 | 451 | 61.0 | 49 | 65.4 |
| HCW in a long-term care facility | 314 | 38.6 | 288 | 39.0 | 26 | 34.6 |
| Unknown | 404 | 17.3 | 329 | 16.4 | 75 | 23.2 |
| **Resident of a long-term care facility** |  |  |  |  |  |  |
| No/unknown | 1867 | 80.3 | 1673 | 83.6 | 194 | 59.9 |
| Yes | 459 | 19.7 | 329 | 16.4 | 130 | 40.1 |
| **City district** |  |  |  |  |  |  |
| Centre | 162 | 7.0 | 141 | 7.0 | 21 | 6.5 |
| New-West | 433 | 18.6 | 360 | 18.0 | 73 | 22.5 |
| North | 268 | 11.5 | 239 | 12.0 | 29 | 9.0 |
| East | 377 | 16.2 | 330 | 16.5 | 47 | 14.5 |
| West | 341 | 14.7 | 293 | 14.6 | 48 | 14.8 |
| South | 350 | 15.1 | 312 | 15.6 | 38 | 11.7 |
| South-East | 355 | 15.3 | 320 | 16.0 | 45 | 10.8 |
| Unknown | 40 | 1.7 | 7 | 0.4 | 33 | 10.2 |
| **Hospitalised** |  |  |  |  |  |  |
| No/unknown | 1730 | 74.4 | 1476 | 73.7 | 254 | 78.4 |
| Yes | 596 | 25.6 | 526 | 26.3 | 70 | 21.6 |
| **Died** |  |  |  |  |  |  |
| No/unknown | 2039 | 87.7 | 1767 | 88.3 | 272 | 84.0 |
| Yes | 287 | 12.3 | 235 | 11.7 | 52 | 16.1 |

**Abbreviations:** IQR, interquartile range

**Table S2. Hospitalisation rates by city district among those linked to the registration database, Amsterdam, the Netherlands, 29 February to 31 May 2020**

|  | **Hospital admissions (n)** | | **Population ^a^** | **Crude rate per 100,000 population (95% CI)** | **Standardised rate per 100,000 population ^b^  (95% CI)** | **Standardised rate difference (95% CI)** | **Standardised rate ratio**  **(95% CI)** |
| --- | --- | --- | --- | --- | --- | --- | --- |
| **Total ^c^** | 523 | 873,055 | | 59.90  (54.88-65.27) |  |  |  |
|  |  |  | |  |  |  |  |
| **City district** |  |  | |  |  |  |  |
| Centre | 29 | 872,23 | | 33.25  (22.27-47.75) | 26.78  (17.89-38.54) | Ref. | Ref. |
| New-West | 109 | 160,115 | | 68.08  (55.90-82.12) | 70.59  (57.93-85.2) | 43.81  (27.29-60.32) | 2.64  (2.22-3.05) |
| North | 79 | 99,705 | | 79.23  (62.73-98.75) | 73.12  (57.72-91.36) | 46.34  (27.31-65.37) | 2.73  (2.30-3.16) |
| East | 58 | 142,011 | | 40.84  (31.01-52.8) | 44.65  (33.82-57.85) | 17.87  (2.69-33.05) | 1.67  (1.22-2.12) |
| West | 74 | 147,831 | | 50.06  (39.31-62.84) | 57.43  (44.99-72.25) | 30.65  (14.20-47.09) | 2.14  (1.71-2.58) |
| South | 72 | 146,471 | | 49.16  (38.46-61.9) | 45.65  (35.65-57.58) | 18.86  (4.41-33.31) | 1.70  (1.27-2.14) |
| South-East | 101 | 89,699 | | 112.60  (91.71-136.82) | 109.92  (89.32-133.85) | 83.14  (59.35-106.92) | 4.10  (3.69-4.52) |

^a^ Population on 1 April 2020

^b^ Standardised for age (in 15-year groups) and gender, using the total population of Amsterdam as the standard population

^c^ 3 matched cases had missing data

**Table S3. Hospitalisation rates by migration history (first and second generation combined) among those linked to the registration database, Amsterdam, the Netherlands, 29 February to 31 May 2020**

|  | **Hospital admissions** | | **Population ^a^** | **Crude rate per 100,000 population (95% CI)** | **Standardised rate per 100,000 population ^b^  (95% CI)** | **Standardised rate difference (95% CI)** | **Standardised rate ratio**  **(95% CI)** |
| --- | --- | --- | --- | --- | --- | --- | --- |
| **Total ^c^** | 523 | 873,055 | | 59.90  (54.88-65.27) |  |  |  |
|  |  |  | |  |  |  |  |
| **Migration background** |  |  | |  |  |  |  |
| Netherlands Antilles | 10 | 12,126 | | 82.47  (39.55-151.66) | 76.75  (36.79-141.19) | 34.71  (-13.24-82.67) | 1.83  (1.19-2.46) |
| Morocco | 67 | 77,213 | | 86.77  (67.25-110.2) | 109.64  (84.08-140.53) | 67.60  (39.71-95.5) | 2.61  (2.32-2.89) |
| Surinam | 78 | 63,944 | | 121.98  (96.42-152.24) | 106.61  (83.8-133.71) | 64.57  (39.69-89.46) | 2.54  (2.27-2.8) |
| Turkey | 41 | 44,417 | | 92.31  (66.24-125.22) | 129.35  (90.48-179.27) | 87.31  (44.51-130.11) | 3.08  (2.72-3.43) |
| Ghana | 17 | 12,883 | | 131.96  (76.87-211.28) | 178.79  (52.99-438.28) | 136.75  (-29.83-303.33) | 4.25  (3.31-5.19) |
| Other non-Western | 49 | 106,137 | | 46.17  (34.15-61.03) | 67.34  (47.20-93.15) | 25.3  (2.58-48.01) | 1.60  (1.25-1.96) |
| Total non-Western | 262 | 316,720 | | 82.72  (73.01-93.37) | 99.08  (87.08-112.28) | 57.05  (43.34-70.75) | 2.36  (2.17-2.54) |
| Total Western | 57 | 169,814 | | 33.57  (25.42-43.49) | 41.06  (30.96-53.4) | -0.98  (-13.28-11.32) | 0.98  (0.68-1.27) |
| Dutch | 204 | 386,521 | | 52.78  (45.78-60.54) | 42.04  (36.38-48.32) | Ref. | Ref. |

^a^ Population on 1 April 2020

^b^ Standardised for age (in 15-year groups) and gender, using the total population of Amsterdam as the standard population

^c^ 3 matched cases had missing data

**Table S4. Hospitalisation rates by migration history (first and second generation combined) among those aged <60 years linked to the registration database, Amsterdam, the Netherlands, 29 February to 31 May 2020**

|  | **Hospital admissions** | | **Population ^a^** | **Crude rate per 100,000 population (95% CI)** | **Standardised rate per 100,000 population ^b^  (95% CI)** | **Standardised rate difference (95% CI)** | **Standardised rate ratio**  **(95% CI)** |
| --- | --- | --- | --- | --- | --- | --- | --- |
| **Total** | 197 | 715,610 | | 27.53 (23.82-31.65) |  |  |  |
|  |  |  | |  |  |  |  |
| **Migration background** |  |  | |  |  |  |  |
| Netherlands Antilles | 3 | 10,044 | | 29.87  (6.16-87.29) | 28.83  (5.94-84.25) | 13.64  (-19.27-46.55) | 1.90  (0.73-3.07) |
| Morocco | 36 | 68,124 | | 52.84  (37.01-73.16) | 59.92  (41.94-83) | 44.74  (24.65-64.82) | 3.95  (3.51-4.38) |
| Surinam | 34 | 49,825 | | 68.24  (47.26-95.36) | 59.79  (40.98-84.26) | 44.6  (23.53-65.67) | 3.94  (3.49-4.39) |
| Turkey | 19 | 39,898 | | 47.62  (28.67-74.37) | 47  (28.28-73.42) | 31.81  (10.22-53.4) | 3.09  (2.56-3.63) |
| Ghana | 7 | 11,056 | | 63.31  (25.46-130.45) | 48.14  (18.95-100.46) | 32.95  (-3.64-69.54) | 3.17  (2.36-3.98) |
| Other non-Western | 30 | 96,978 | | 30.93  (20.87-44.16) | 33.38  (22.39-47.89) | 18.2  (5.32-31.07) | 2.20  (1.74-2.66) |
| Total non-Western | 129 | 275,925 | | 46.75  (39.03-55.55) | 48.3  (40.32-57.39) | 33.11  (23.71-42.52) | 3.18  (2.85-3.52) |
| Total Western | 21 | 146,910 | | 14.29  (8.85-21.85) | 14.83  (9.12-22.79) | -0.36  (-8.12-7.40) | 0.98 (0.46-1.5) |
| Dutch | 47 | 292,775 | | 16.05  (11.8-21.35) | 15.19  (11.14-20.22) | Ref. | Ref. |

^a^ Population on 1 April 2020

^b^ Standardised for age (in 15-year groups) and gender, using the total population of Amsterdam as the standard population

**Table S5. Hospitalisation rates by migration history (first and second generation combined) among those aged ≥60 years linked to the registration database, Amsterdam, the Netherlands, 29 February to 31 May 2020**

|  | **Hospital admissions** | | **Population ^a^** | **Crude rate per 100,000 population (95% CI)** | **Standardised rate per 100,000 population ^b^  (95% CI)** | **Standardised rate difference (95% CI)** | **Standardised rate ratio**  **(95% CI)** |
| --- | --- | --- | --- | --- | --- | --- | --- |
| **Total** | 326 | 157,445 | | 207.06  (185.19-230.8) |  |  |  |
|  |  |  | |  |  |  |  |
| **Migration background** |  |  | |  |  |  |  |
| Netherlands Antilles | 7 | 2,082 | | 336.22  (135.18-692.73) | 221.84  (89.13-457.24) | 34.03  (-133.26-201.32) | 1.18  (0.42-1.94) |
| Morocco | 31 | 9,089 | | 341.07  (231.74-484.12) | 369.25  (235.99-550.51) | 181.44  (29.8-333.09) | 1.97  (1.53-2.4) |
| Surinam | 44 | 14,119 | | 311.64  (226.44-418.36) | 309.29  (212.07-435.76) | 121.49  (10.58-232.39) | 1.65  (1.27-2.03) |
| Turkey | 22 | 4,519 | | 486.83  (305.1-737.07) | 537.6  (306.7-874.28) | 349.8  (83.8-615.8) | 2.86  (2.34-3.38) |
| Ghana | 10 | 1,827 | | 547.35  (262.47-1006.59) | 993.63  (94.21-3901.41) | 805.83  (-675.28-2286.93) | 5.29  (3.79-6.79) |
| Other non-Western | 19 | 9,159 | | 207.45  (124.9-323.95) | 256.7  (127.5-460.89) | 68.89  (-86.81-224.59) | 1.37  (0.75-1.98) |
| Total non-Western | 133 | 40,795 | | 326.02  (272.97-386.37) | 344.08  (278.02-421.12) | 156.27  (80.16-232.39) | 1.83  (1.57-2.09) |
| Total Western | 36 | 22,904 | | 157.18  (110.09-217.6) | 180.69  (122.95-256.16) | -7.12  (-77.59-63.35) | 0.96  (0.57-1.35) |
| Dutch | 157 | 93,746 | | 167.47  (142.3-195.82) | 187.81  (158.26-221.27) | Ref. | Ref. |

^a^ Population on 1 April 2020

^b^ Standardised for age (in 15-year groups) and gender, using the total population of Amsterdam as the standard population

**Table S6. Associations with COVID-19 related hospitalisation, obtained from a Poisson model with an interaction term between migration background and city district, Amsterdam, the Netherlands, 29 February to 31 May 2020**

| **Factor** | **RR (95% CI)** | ***P*-value** |
| --- | --- | --- |
| **Migration background** |  |  |
| None (ethnic-Dutch) | 1 |  |
| Western | 0.74 (0.47-1.11) | 0.16 |
| Non-Western | 2.33 (1.77-3.06) | <0.001 |
|  |  |  |
| **City district** |  |  |
| Central (C/W/S/E) | 1 |  |
| Peripheral (SE/N/NW) | 1.49 (1.13-1.96) | 0.005 |
|  |  |  |
| **Migration background * district term** | |  |
| Western/Peripheral | 1.78 (0.99-3.24) | 0.06 |
| Non-Western/Peripheral | 0.92 (0.63-1.34) | 0.66 |
|  |  |  |
| **Sex** |  |  |
| Male | 1 |  |
| Female | 0.59 (0.49-0.7) | <0.001 |
|  |  |  |
| **Age** |  |  |
| <45 years | 1 |  |
| 45-59 years | 4.96 (3.74-6.61) | <0.001 |
| 60-74 years | 13.49 (10.45-17.59) | <0.001 |
| ≥75 years | 23.63 (17.75-31.62) | <0.001 |

Likelihood ratio test *P*-value this model vs. model without interaction term = 0.08

**Table S7. Associations with COVID-19 related hospitalisation, obtained from a Poisson model with an interaction term between migration background and age, Amsterdam, the Netherlands, 29 February to 31 May 2020**

| **Factor** | **RR (95% CI)** | ***P*-value** |
| --- | --- | --- |
| **Migration background** |  |  |
| None (ethnic-Dutch) | 1 |  |
| Western | 1.23 (0.54-2.71) | 0.61 |
| Non-Western | 3.28 (1.90-6.03) | <0.001 |
|  |  |  |
| **City district** |  |  |
| Central (C/W/S/E) | 1 |  |
| Peripheral (SE/N/NW) | 1.50 (1.26-1.79) | <0.001 |
|  |  |  |
| **Sex** |  |  |
| Male | 1 |  |
| Female | 0.58 (0.49-0.69) | <0.001 |
|  |  |  |
| **Age** |  |  |
| <45 years | 1 |  |
| 45-59 years | 6.02 (3.32-11.44) | <0.001 |
| 60-74 years | 17.95 (10.69-32.37) | <0.001 |
| ≥75 years | 36.13 (21.36-65.46) | <0.001 |
|  |  |  |
| **Migration * age term** |  |  |
| Western/45-59 years | 0.76 (0.26-2.20) | 0.62 |
| Non-Western/45-59 years | 0.79 (0.38-1.57) | 0.51 |
| Western/60-74 years | 0.79 (0.31-2.06) | 0.63 |
| Non-Western/60-74 years | 0.68 (0.35-1.27) | 0.24 |
| Western/≥75 years | 0.79 (0.30-2.11) | 0.63 |
| Non-Western/≥75 years | 0.45 (0.21-0.89) | 0.026 |

Likelihood ratio test *P*-value this model vs. model without interaction term = 0.33

**Table S8. Associations with COVID-19 related hospitalisation, obtained from a Poisson model with an interaction term between migration background and sex, Amsterdam, the Netherlands, 29 February to 31 May 2020**

| **Factor** | **RR (95% CI)** | ***P*-value** |
| --- | --- | --- |
| **Migration background** |  |  |
| None (ethnic-Dutch) | 1 |  |
| Western | 1.11 (0.76-1.58) | 0.59 |
| Non-Western | 2.20 (1.73-2.79) | <0.001 |
|  |  |  |
| **City district** |  |  |
| Central (C/W/S/E) | 1 |  |
| Peripheral (SE/N/NW) | 1.53 (1.28-1.82) | <0.001 |
|  |  |  |
| **Sex** |  |  |
| Male | 1 |  |
| Female | 0.60 (0.46-0.80) | <0.001 |
|  |  |  |
| **Migration * sex term** |  |  |
| Western/Female | 0.69 (0.36-1.28) | 0.25 |
| Non-Western/Female | 1.01 (0.70-1.47) | 0.95 |
|  |  |  |
| **Age** |  |  |
| <45 years | 1 |  |
| 45-59 years | 4.95 (3.73-6.60) | <0.001 |
| 60-74 years | 13.53 (10.48-17.64) | <0.001 |
| ≥75 years | 23.85 (17.93-31.90) | <0.001 |

Likelihood ratio test *P*-value this model vs. model without interaction term = 0.45
